# The BBN model: a mouse bladder cancer model featuring basal-subtype gene expression and MLL3/MLL4 genetic disruption

**DOI:** 10.18632/oncoscience.439

**Published:** 2018-06-29

**Authors:** Damiano Fantini, Joshua J. Meeks

**Affiliations:** Department of Urology, Northwestern University, Feinberg School of Medicine, Chicago, IL 60611, USA; Robert H. Lurie Comprehensive Cancer Center, Northwestern University, Chicago, IL 60611, USA; Department of Biochemistry and Molecular Genetics, Feinberg School of Medicine Northwestern University, Chicago, IL 60611, USA

**Keywords:** bladder cancer, cancer genomics, cancer models, BBN tumors, epigenetic factors

Bladder cancer is one of the most common cancers in the United States, with an incidence of more than 80,000 new cases per year. The major risk factors for bladder cancer include cigarette smoking, as well as gender, since men are 4 times more likely than women to develop urothelial carcinomas [1]. About 30% of new patients are diagnosed with muscle-invasive bladder cancer (MIBC), with an unfavorable prognosis compared to non-muscle invasive tumors, and only few therapeutic options. Currently, a major issue limiting the development of more effective anticancer drugs for MIBC is the small number of animal models that closely recapitulate the human disease. A promising MIBC animal model is the N-butyl-N-(4-hydroxybutyl)-nitrosamine (BBN) mouse model [2]. BBN is a carcinogen structurally related to the chemicals found in cigarette smoke. When administered to C57/B6 male mice in drinking water for at least 20 weeks, BBN causes invasive bladder tumors. Early studies revealed morphologic similarities between murine BBN tumors and human bladder tumors. Studies by The Cancer Genome Atlas (TCGA) suggested that MIBC can be further classified into distinct molecular subtypes independently of morphology, incorporating information about genomic alterations and gene expression dysregulations [3]. Notably, cancer molecular subtypes are prognostic of survival, predict sensitivity to specific therapies (chemotherapy and immunotherapy), and hence may aid in precision medicine. To better understand whether the BBN mouse bladder cancer model closely mimicked a specific subgroup of human MIBC, we analyzed its molecular alterations by RNA-seq and whole exome sequencing (WES) [2].

Our RNA-seq analyses revealed that gene expression in the BBN tumors aligned to MIBC belonging to the basal molecular subtype, with increased expression of markers such as *Cd44*, *Cdh3*, *Krt5*, and *Krt14*. In addition, we observed dysregulation of genes associated with T-cell homeostasis (*Il7r*, *Il2ra*, *Ripk3*) and extracellular signaling [2]. These observations were suggestive of an active but ineffective response of the murine immune system to the BBN-induced tumors. While we are still conducting experiments to validate this hypothesis, our observations suggested that the BBN model could be a valid system to test immune checkpoint inhibition, or to develop anticancer drugs aimed at modulating the immune system.

Our WES analyses revealed that the BBN tumors accumulated somatic mutations at rates comparable with those reported for human MIBC [3]. We identified a panel of genes frequently mutated in both human and mouse bladder tumors, including *Trp53* (*P53*, BBN mutation rate = 80%), *Kmt2c* (*MLL3*, BBN mutation rate = 90%), and *Kmt2d* (*MLL4*, BBN mutation rate = 70%) [2]. A closer inspection of these three genes revealed that mouse tumors accumulated mutations that matched global or local hotspot mutations found in the coding sequences of the corresponding human orthologs. This supports the hypothesis that *P53*, *MLL3*, and *MLL4* play driver roles in bladder tumorigenesis or cancer progression in both human and murine tumors. Notably, both *MLL3* and *MLL4* encode for enzymes belonging to the methyltransferase family, and are involved in the epigenetic regulation of enhancer activity via methylation of histone *H3K4* [4]. Our findings proved that genetic aberrations of chromatin remodeling processes and other epigenetic factors may be crucial in both human and mouse bladder tumors. Notably, we did not detect any mutation in *Kdm6a*, a histone demethylase which is frequently mutated in human MIBC (TCGA mutation rate = 24%) [2]. We analyzed human TCGA bladder tumors, and found that *KDM6A* mutations were mutually exclusive with *MLL4*. Consistently, in BBN tumors the lack of *Kdm6a* mutation was accompanied by *Kmt2d* mutations in most tumor samples. Additionally, independent reports revealed that *KDM6A* mutations were enriched in low-grade low-stage luminal bladder tumors [4], supporting that the BBN bladder cancer model is a good model of high-grade basal-like MIBC [2].

We also extracted two mutational signatures (MOUSIG-A, and MOUSIG-B) from the BBN cancer genomes [5]. Mutational signatures are discrete patterns of tri-nucleotide mutation types (single-nucleotide variants and their flanking nucleotides) contributing to genetic instability in cancer. All BBN tumors included comparable levels of MOUSIG-A-associated mutations [2]. This signature matched to the human COSMIC-5 signature, previously linked to defects in the nucleotide excision DNA repair pathway [6]. MOUSIG-A may be the direct result of the activity of BBN on the murine urothelium. Conversely, MOUSIG-B signature was found in BBN tumors at variable levels, may be the result of a complex set of genomic aberrations, and featured high levels of T>A mutations [2]. Notably, we could not detect APOBEC mutational patterns in the BBN tumors, which on the contrary are prevalent in human tumors [7, 8]. While this finding is likely the result of genetic differences between humans and mice (humans express 7 APOBEC3 isoforms, mice only one), it also suggests that the APOBEC mutational processes may not be reproduced in a BBN model that uses wild type C57/B6 animals [2].

**Figure 1:**
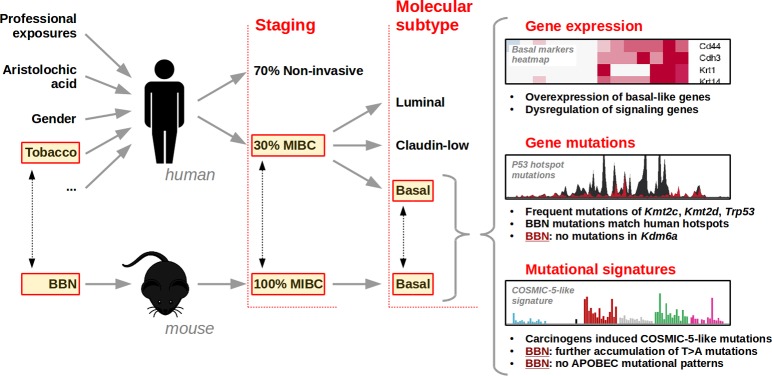
Diagram summarizing the different risk factors and exposures responsible for the development of bladder cancer in humans and mice The different types of bladder cancer (with respect to staging and molecular subtypes) are illustrated. The main similarities and differences in terms of gene expression, gene mutations, and mutational signatures are highlighted.

Together, our study showed that the BBN model mimics very well human high-grade basal-like MIBC (Figure 1), and paved the way for using this model in studies of bladder cancer progression and drug discovery.
